# A machine learning strategy for predicting localization of post-translational modification sites in protein-protein interacting regions

**DOI:** 10.1186/s12859-016-1165-8

**Published:** 2016-08-17

**Authors:** Thammakorn Saethang, D. Michael Payne, Yingyos Avihingsanon, Trairak Pisitkun

**Affiliations:** 1 Systems Biology Center, Research Affairs, Faculty of Medicine, Chulalongkorn University, 1873 Rama 4 Road, Pathumwan, Bangkok, 10330 Thailand; 2 Department of Medicine, Division of Nephrology, Faculty of Medicine, Chulalongkorn University, 1873 Rama 4 Road, Pathumwan, Bangkok, 10330 Thailand; 3 Epithelial Systems Biology Laboratory, NHLBI, National Institutes of Health, Bethesda, MD 20892-1603 USA

**Keywords:** Post-translational modification, Protein-protein interacting region, Machine learning, AAindex

## Abstract

**Background:**

One very important functional domain of proteins is the protein-protein interacting region (PPIR), which forms the binding interface between interacting polypeptide chains. Post-translational modifications (PTMs) that occur in the PPIR can either interfere with or facilitate the interaction between proteins. The ability to predict whether sites of protein modifications are inside or outside of PPIRs would be useful in further elucidating the regulatory mechanisms by which modifications of specific proteins regulate their cellular functions.

**Results:**

Using two of the comprehensive databases for protein-protein interaction and protein modification site data (PDB and PhosphoSitePlus, respectively), we created new databases that map PTMs to their locations inside or outside of PPIRs. The mapped PTMs represented only 5 % of all known PTMs. Thus, in order to predict localization within or outside of PPIRs for the vast majority of PTMs, a machine learning strategy was used to generate predictive models from these mapped databases. For the three mapped PTM databases which had sufficient numbers of modification sites for generating models (acetylation, phosphorylation, and ubiquitylation), the resulting models yielded high overall predictive performance as judged by a combined performance score (CPS). Among the multiple properties of amino acids that were used in the classification tasks, hydrophobicity was found to contribute substantially to the performance of the final predictive models. Compared to the other classifiers we also evaluated, the SVM provided the best performance overall.

**Conclusions:**

These models are the first to predict whether PTMs are located inside or outside of PPIRs, as demonstrated by their high predictive performance. The models and data presented here should be useful in prioritizing both known and newly identified PTMs for further studies to determine the functional relationship between specific PTMs and protein-protein interactions. The implemented R package is available online (http://sysbio.chula.ac.th/PtmPPIR).

**Electronic supplementary material:**

The online version of this article (doi:10.1186/s12859-016-1165-8) contains supplementary material, which is available to authorized users.

## Background

Post-translational modification (PTM) of proteins is a key mechanism for cellular regulation including protein-protein interactions, protein functions, protein turnover, protein localization, cell signaling, and proteomic diversity [1, 2]. More than 200 different types of amino acid-specific PTMs have been identified, including acetylation, methylation, glycosylation, phosphorylation, sumoylation, ubiquitylation and so on [2]. Several types of PTMs are known to have specific functions regarding protein-protein interactions: for example, phosphorylation sites tend to be localized on protein binding hotspots and modulate the stability of protein interactions [3]; ubiquitylation plays an important role in cellular signaling such as protein degradation, autophagy, and protein turnover by promoting interactions with various proteins which recognize this PTM [4–6]; acetylation controls a variety of cellular processes, and alters the properties of protein-binding interfaces by neutralizing the positive charge of the lysine residues or disrupting hydrogen bonds on lysine side-chains [7].

Because of advances in high-throughput technologies especially in protein mass spectrometry, enormous amounts of data related to PTMs have been obtained. At the present, there are multiple databases available for studying PTMs such as UniProt [8], dbPTM [9], PTMCuration [10], PTMcode [11], and PhosphoSitePlus [12]. Among these databases, PhosphoSitePlus is the largest, most frequently updated and curated PTM database which both stores non-redundant information and provides tools for studying PTMs [9, 12, 13]. While the rate of new PTM identification is rapid, the functional annotation process of these PTMs is relatively slow. Functional annotation of PTM sites is usually obtained from different experimental methods, e.g., site-directed mutagenesis, radiolabeling, immunoblot analysis, and multidimensional liquid chromatography tandem mass spectrometry [14]. However, these methods typically take a long time to implement. Computational methods that can help to predict the functional significance of new PTM sites will allow researchers to prioritize their targets for the functional validation. Several attributes of PTM sites, such as the degree of conservation and the localization in functional domains, have been used for such predictions [9, 10, 14]. One of the important functional domains on proteins is a protein-protein interacting region (PPIR), which is a binding/interacting interface between a protein and its protein partner/substrate. PTMs that occur in the PPIR can either interfere with or facilitate the interaction between proteins, thus they are functionally important.

PPIRs can only be confidently identified from protein structures that were determined using high resolution techniques such as X-ray crystallography, NMR spectroscopy, and/or cryo-electron microscopy. The Protein Data Bank (PDB) is a rapidly expanding resource consisting of a large number of structures for protein-protein complexes, including detailed information of PPIRs at the amino acid residue level [15]. These structures can be represented as 3D images and enable the interacting regions of proteins to be visualized and identified. The integration of this structural information with PTM identification could greatly facilitate the determination of the functional relationship between specific PTMs and protein-protein interactions.

We began this study with a simple question: For any detected protein modification, can we predict whether that modification is inside or outside of a PPIR? To address this question, we first integrated the information from the PDB and PhosphoSitePlus databases to generate new supervised datasets indicating which PTM sites are experimentally confirmed to be inside or outside of PPIRs (mapped PTMs). Subsequently, we used several conventional features including hydropathy index, secondary structure, PSSM (Position-Specific Scoring Matrix), and sequence conservation (surrounding the modified site), along with existing web-based applications, to perform the prediction. However, these features and applications proved to be insufficient to accomplish the task. Therefore, a different approach was required.

One strategy that has been used successfully to make predictions based on pattern recognition is “machine learning”. In fact, this strategy has been recently employed to predict individual sites of protein modification with high performance [16–18], although with no indication of whether the modified sites are inside or outside of PPIRs. Therefore, we applied a machine learning strategy to generate the models for predicting whether a known PTM site is inside or outside of a PPIR. Since most machine learning algorithms require numeric data, the mapped PTM-specific sequence datasets from PhosphoSitePlus database were first encoded numerically using AAindex [19], a database of numerical indices representing physicochemical and biochemical properties of amino acids, which has been utilized in numerous previous applications for decades. For example, the AAindex database was recently used in a machine learning strategy to develop the PAAQD [20] and EpicCapo [21] applications for immunogenicity and epitope predictions, respectively. The performance of PAAQD and EpicCapo was high and outperformed other applications at the time they were developed, supporting the use of both the AAindex database and a machine learning strategy for predictive modeling.

In this study, the integrated PDB/PTM-specific datasets were analyzed and modeled using machine learning algorithms. Our predictive models showed high performance measures, and important features contributing to predictive power were identified. These predictive models are available online and may be useful in providing additional insight related to the functional relationship between specific PTMs and protein-protein interactions.

## Results

### Preparation of datasets for generating predictive models

**Fig. 1:**
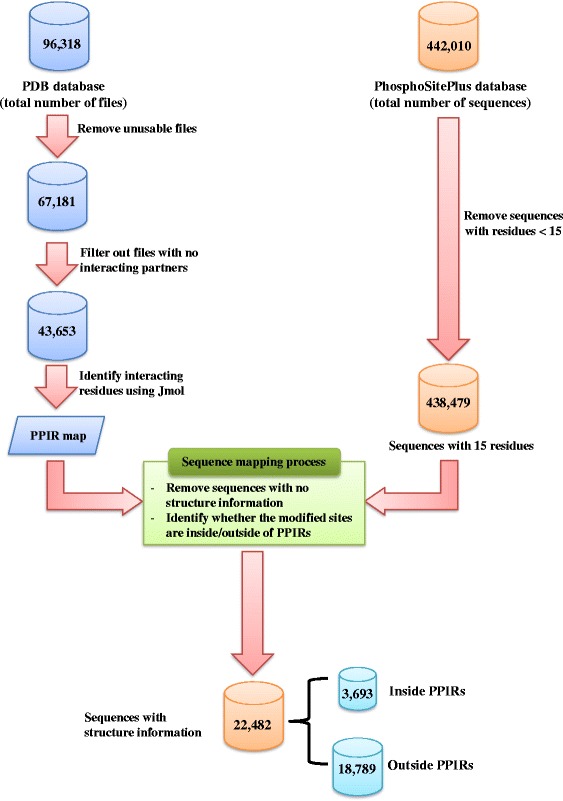
Flow diagram of the data generation and preparation processes. Numerical results are shown for each step of the overall process

**Fig. 2:**
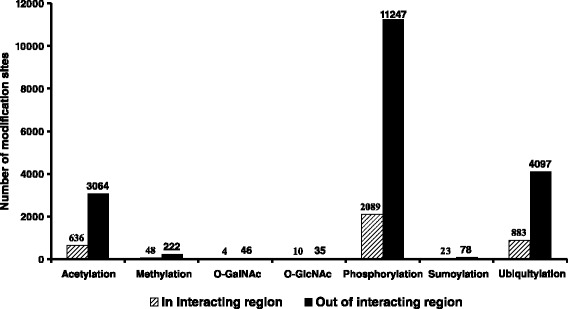
The numbers of modification sites inside and outside of PPIRs

Figure 1 illustrates the workflow and overall results of the data generation and preparation processes. Starting with the entire PDB database, filtering steps were employed to generate a dataset containing only structures for proteins with interacting protein partners. The number of PDB files was decreased by slightly more than half during the filtering processes. For the PhosphoSitePlus database, entries are provided as sequences with lengths up to 15 amino acid residues, with up to ±7 neighboring AAs surrounding the PTM site. After removing sequences with lengths < 15 residues (i.e., with modification sites close to protein termini, which represented less than 1 % of sequences), the number of remaining sequences was further dramatically reduced (by 95 %) following the sequence mapping process. The distribution of modification sites inside and outside of PPIRs for each individual type of PTM is shown in Fig. 2.

Although the number of data points required for machine learning is a function of the variability and complexity of the datasets and research problems being addressed, at least 75–80 data points have been reported as the minimum necessary for achieving acceptable performance levels [22, 23]. This suggests that a total of approximately 500 data points might be needed to generate reliable predictive models using 5-fold cross-validation as employed in this study. Consequently, only the three datasets for acetylation, phosphorylation, and ubiquitylation included enough sequences to be used for further analyses.

### Use of conventional features to generate predictive models

Initially, we focused on the phosphorylation dataset and performed position-specific sequence analysis surrounding the phosphorylation sites found either within or outside of PPIRs using PhosphoLogo [24].

**Fig. 3:**
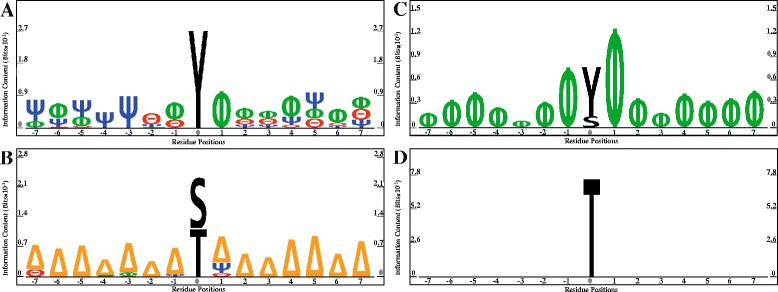
Results of PhosphoLogo analysis. The x- and y-axes correspond to residue positions and bit scores (×10^−1^), respectively. For phosphorylated sequences within PPIRs, position-specific sequence analyses revealed favored (**a**, logo) and disfavored (**b**, anti-logo) amino acid residues. Similar analyses of phosphorylated sequences outside PPIRs were performed (**c**, logo, and **d**, anti-logo). Amino acid types for neighboring positions of central phosphorylated residues (S, T, Y, or H) are indicated by symbols as follows: Φ = nonpolar, Δ = polar, Θ = acidic, Ψ = basic

**Fig. 4:**
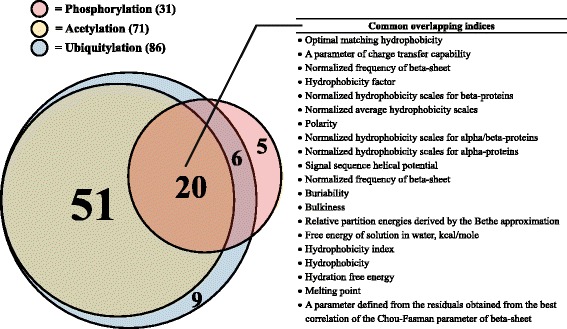
Overlap among of the three PTM-specific optimized sets of indices. Reference for each index is showed in Additional file 14: Table S6

Using this conventional approach, a few general conclusions were possible: 1) there was a rather strong preference for tyrosine as the phosphorylation site within PPIRs (Fig. 3a); 2) the identity of amino acid residues surrounding the phosphorylation site within PPIRs was not completely random, revealing a preference for certain amino acid properties at some specific positions (Fig. 3a); 3) the anti-logo analysis showed that serine and threonine were strongly disfavored as the phosphorylation site within PPIRs (Fig. 3b); 4) the anti-logo analysis also demonstrated that polar amino acids were strongly disfavored at all positions surrounding the phosphorylation site within PPIRs (Fig. 3b); and 5). Finally, nonpolar amino acids were favored for all positions surrounding the phosphorylation site outside PPIRs (Fig. 3c), and no preference was detected for amino acid residues surrounding that phosphorylation site following anti-logo analysis (Fig. 3d). While this analysis revealed some possible patterns, no quantitative rules were generated that would enable development of a prediction tool. Furthermore, when the same type of analysis was performed on ubiquitylation and acetylation datasets using Motif-x [25], no pattern of preferences was detected.

Next, we used individual conventional features (namely, amino acid hydropathy, secondary structure, and homolog site/motif conservation) in a machine learning strategy in an attempt to develop models for predicting whether modification sites are inside or outside of PPIRs (see Methods for details). Table 1 shows the final results for the initial machine learning strategy using these three conventional features. Obviously, the predictive performance was poor, essentially being no better than random chance. Therefore, we developed an alternate machine learning strategy based on encoding a large number of features representing various physicochemical and biochemical properties of amino acids.

**Table 1:** Classification results for each PTM-specific dataset using conventional features and the SVM as a classifier^a^

	F_1_	S_n_	S_p_	PPV	ACC	AUC	MCC
Acetylation							
Hydropathy	0.51	0.51	0.51	0.51	0.51	0.50	0.01
Secondary structure	0.49	0.49	0.50	0.49	0.49	0.50	−0.01
Conservation	0.55	0.57	0.48	0.53	0.53	0.54	0.06
Combined features	0.53	0.53	0.54	0.53	0.54	0.55	0.07
Phosphorylation							
Hydropathy	0.53	0.58	0.40	0.49	0.49	0.48	−0.03
Secondary structure	0.48	0.45	0.58	0.52	0.52	0.53	0.03
Conservation	0.53	0.55	0.45	0.50	0.50	0.51	0.01
Combined features	0.55	0.56	0.52	0.54	0.54	0.55	0.08
Ubiquitylation							
Hydropathy	0.52	0.53	0.49	0.51	0.51	0.52	0.02
Secondary structure	0.48	0.46	0.52	0.49	0.49	0.48	−0.02
Conservation	0.57	0.59	0.50	0.54	0.55	0.56	0.09
Combined features	0.54	0.54	0.53	0.53	0.53	0.55	0.06

^a^This table shows results when datasets were balanced (see Methods). The results using unbalanced datasets are shown in Additional file 7: Table S1

### Initial predictive models for PTM-specific datasets using multiple features

Three PTM-specific datasets (i.e., acetylation, phosphorylation, and ubiquitylation) were individually encoded into numerical arrays using 102 indices of AAindex1 [19] and then analyzed using the SVM, with 10 independent iterations of 5-fold cross-validation to evaluate for classification performance measures.

**Fig. 5:**
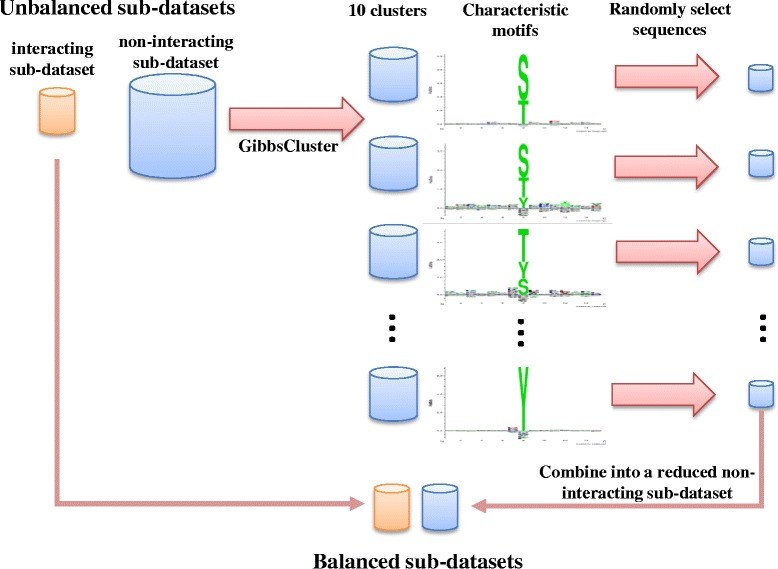
The sampling strategy for balancing interacting and non-interacting sub-datasets. The larger non-interacting sub-dataset was clustered by GibbsCluster into 10 clusters. Each cluster contained sequences representing a different characteristic motif; for the purpose of illustration, shown here are example motifs from the phosphorylation dataset. Equal numbers of sequences from each cluster were randomly selected and combined to create a reduced non-interacting sub-dataset which was similar in size to the interacting sub-dataset

As shown in Fig. 2, all PTM-specific datasets were imbalanced, i.e., the number of modification sites outside of PPIRs is substantially greater than that of sites inside of PPIRs. As we expected, by using all data without correcting for imbalance, S_n_, AUC, and MCC were low (<0.5), while S_p_ could reach the maximum at 1 (Additional file 1: Table S2). Thus, it was necessary to correct for imbalance before proceeding to the model generation process, and the GibbsCluster [26] clustering algorithm was employed in the balancing process (see Methods and Fig. 5). Subsequently, the balanced datasets were analyzed in classification tasks using the SVM as the classifier; the results are shown in Table 2. The performance measures are shown as averaged values of 10 iterations of 5-fold cross-validation. Overall, the resulting performance measures were quite good, with minimum values greater than 0.70 and most values greater than 0.85. This indicated that the predictive power of our machine learning strategy was relatively robust.

**Table 2:** Averaged performance measures for each PTM-specific dataset using the SVM as a classifier and 102 indices of AAindex1 in the encoding process

Dataset	F_1_	S_n_	S_p_	PPV	ACC	AUC	MCC
Acetylation	0.86	0.81	0.94	0.93	0.87	0.89	0.75
Phosphorylation	0.84	0.74	0.99	0.99	0.86	0.92	0.75
Ubiquitylation	0.86	0.81	0.92	0.92	0.86	0.90	0.73

The highest possible value for all measures is 1

Standard deviations for all values were < ±0.005

### Predictive model refinement by feature selection

In order to improve the power of the predictive models and to eliminate indices making no contribution to the predictive power, the datasets were analyzed using individual indices which were then combined one at a time in an iterative process. For each encoded PTM-specific dataset, the results using the SVM for each individual index were ranked based on CPS (i.e., summation of ACC, MCC, and AUC; see Methods). Classification tasks were then repeated by consecutive addition of each index-specific sub-dataset in order of rank until an optimal set of indices was identified, based on reaching a maximum value for CPS. This rank based optimization uncovered and preserved information related to the relative importance of each index in contributing to the predictive power of the models.

Table 3 shows the result of classification when only sub-datasets corresponding to indices in the optimal sets were used. From the total of 102 indices in AAindex1, the numbers of indices after optimization were decreased to 71, 31, and 86 for acetylation, phosphorylation, and ubiquitylation, respectively. For acetylation and phosphorylation, many of the performance measures were significantly increased after optimization when compared with the use of all 102 indices. However, except for MCC, the performance measures were unchanged for the ubiquitylation dataset. Figure 4 shows the overlap of optimal sets of indices for the PTM-specific datasets, including the 20 indices that were common to all three datasets after optimization. Interestingly, 11 of the 20 indices were related to the same basic property of amino acids, namely hydrophobicity. In addition, all 71 of the indices from the optimized acetylation-specific dataset were also present in the optimized ubiquitylation-specific dataset. The full list of indices included in the optimized sets is shown in Additional file 2: Table S3, Additional file 3: Table S4, Additional file 4: Table S5.

**Table 3:** Resulting performance measures when only sub-datasets corresponding to indices in the optimal sets were used in the classification tasks

Dataset	F_1_	S_n_	S_p_	PPV	ACC	AUC	MCC	#indices used (of 102 total)	#features used (of 1428 total)
Acetylation	0.87^a^	0.82	0.93	0.93	0.87	0.90^a^	0.76^a^	71	994
Phosphorylation	0.89^a^	0.81^a^	1.00	0.99	0.90^a^	0.92	0.82^a^	31	434
Ubiquitylation	0.86	0.81	0.92	0.93^a^	0.87	0.90	0.74^a^	86	1204

The maximum possible value for all measures is 1

^a^Significantly increased (*t*-test, α < 0.05) when compared with the use of all 102 indices

For further refinement of the predictive models, Relief-F [27] and Information Gain [28] algorithms were employed to rank the features. The best-feature subsets were constructed by including the features sequentially, one by one, from the top ranked feature to the last one in the classification task using the SVM (see Methods). Table 4 shows the resulting performance measures when best-feature subsets were used in the classification tasks. ACC, AUC, and MCC were comparable for both feature selection algorithms, and were significantly increased compared with the performance measures without feature selection. In addition, both algorithms dramatically reduced the number of features required for generating predictive models, with Information Gain requiring only about half as many features as Relief-F.

**Table 4:** Resulting performance measures when best-feature sets were used in the classification tasks, using two different feature selection algorithms

Dataset	Relief-F						
	ACC	AUC	MCC	PPV	S_n_	S_p_	#Features used
Acetylation	0.88	0.92	0.78	0.95	0.82^a^	0.95	144
Phosphorylation	0.91	0.93	0.83	0.99^a^	0.82	1.00^a^	73
Ubiquitylation	0.88	0.91	0.77	0.96	0.80^a^	0.96	512
	Information Gain						
Acetylation	0.88	0.90^a^	0.78	0.97	0.80^a^	0.97	82
Phosphorylation	0.91	0.93	0.84	0.99^a^	0.83	1.00^a^	35
Ubiquitylation	0.88	0.91	0.77	0.96	0.80^a^	0.96	343

^a^Except for these values, others were significantly increased (*t*-test, α < 0.05) when compared with the results using optimized sets of indices shown in Table 3

### Comparisons of classifiers

For the predictive models described in the preceding sections, the SVM was used as a classifier. Next, using the optimized lists of indices and features identified in previous section as an input, we evaluated the performance of five additional classifiers: k-nearest neighbors (k-NN), Random Forest (RF), C4.5, KStar, and Multilayer Perceptron (MLP). Table 5 compares the resulting performance measures of all six classifiers for all three PTM-specific datasets.

**Table 5:** Resulting performance measures of all six classifiers for all three PTM-specific datasets, using the optimized lists of indices and features as an input

Classifier	Dataset	Relief-F				Information gain			
		ACC	AUC	MCC	CPS	ACC	AUC	MCC	CPS
SVM	Acetylation	0.88	0.92	0.78	2.58	0.88	0.90	0.78	2.56
	Phosphorylation	0.91	0.93	0.83	2.67	0.91	0.93	0.84	2.68
	Ubiquitylation	0.88	0.91	0.77	2.56	0.88	0.91	0.77	2.56
		summation			7.81	summation			7.80
k-NN	Acetylation	0.87	0.91	0.74	2.52	0.87	0.91	0.75	2.53
	Phosphorylation	0.89	0.93	0.80	2.62	0.92	0.93	0.84	2.69
	Ubiquitylation	0.80	0.86	0.61	2.27	0.81	0.89	0.65	2.35
		summation			7.41	summation			7.57
RF	Acetylation	1.00	1.00	1.00	3.00	1.00	1.00	1.00	3.00
	Phosphorylation	0.91	0.93	0.82	2.66	0.90	0.93	0.80	2.63
	Ubiquitylation	1.00	1.00	1.00	3.00	1.00	1.00	1.00	3.00
		summation			8.66	summation			8.63
C4.5	Acetylation	0.85	0.87	0.70	2.42	0.88	0.89	0.76	2.53
	Phosphorylation	0.89	0.90	0.78	2.57	0.92	0.93	0.85	2.70
	Ubiquitylation	0.80	0.82	0.61	2.23	0.81	0.82	0.62	2.25
		summation			7.22	summation			7.48
KStar	Acetylation	0.83	0.88	0.65	2.36	0.83	0.89	0.66	2.38
	Phosphorylation	0.87	0.92	0.74	2.53	0.89	0.93	0.79	2.61
	Ubiquitylation	0.71	0.76	0.43	1.90	0.79	0.82	0.57	2.18
		summation			6.79	summation			7.17
MLP	Acetylation	0.85	0.91	0.70	2.46	0.84	0.90	0.67	2.41
	Phosphorylation	0.88	0.92	0.76	2.56	0.91	0.93	0.83	2.67
	Ubiquitylation	0.84	0.90	0.68	2.42	0.83	0.89	0.66	2.38
		summation			7.44	summation			7.46

Except for RF (see below), the SVM provided the best performance overall (i.e., for the entire collection of PTM data, based on total sum of CPS values), and the best individual performance for two of the three PTM-specific datasets, namely acetylation and ubiquitylation. For the phosphorylation-specific dataset, the k-NN and C4.5 classifiers (with Information Gain algorithm) provided slightly better performance than the SVM. Despite the latter results, since the SVM was used to obtain the optimized lists of indices and features for subsequent modeling by the other classifiers, some biases may have been introduced (e.g., ranking order, indices/features excluded, etc.) that compromised the performance of the other classifiers. To address this issue, the phosphorylation-specific dataset was re-evaluated using either k-NN or C4.5 exclusively as the classifiers (instead of the SVM) for all feature selection and classification tasks. As shown in Table 6, the performance measures using the optimized lists of indices and features identified by k-NN or C4.5 were both lower than those using the SVM. This indicated that no significant bias in the refinement processes was introduced when the SVM was used. Finally, while the performance of RF appeared to be “perfect” (maximum of 1.00 for all three measures) for the acetylation and ubiquitylation datasets, this result is clearly an anomaly, resulting from over-fitting [29]. Over-fitting as a result of using RF was not surprising, since previous experiments showed that the performance of RF implemented in Weka frequently outperformed other classifiers, but the resulting predictive models using RF tend to be over-fitted [29].

**Table 6:** Resulting performance measures for the phosphorylation dataset, using k-NN or C4.5 as the classifiers (instead of the SVM) for all feature selection and classification tasks, including identification of optimized sets of indices and features

Classifier	Dataset	Information gain						
		PPV	S_n_	S_p_	ACC	AUC	MCC	CPS
SVM	Phosphorylation	0.99	0.83	1.00	0.91	0.93	0.84	2.68
k-NN	Phosphorylation	0.95	0.85	0.95	0.89	0.92	0.79	2.60
C4.5	Phosphorylation	0.96	0.87	0.97	0.91	0.92	0.84	2.67

### Implementation and evaluation of final predictive models

In order to maximize performance and minimize the number of features required, the final PTM-specific predictive models were generated using the optimized lists of indices and features as described (Tables 3 and 4), employing Relief-F for the acetylation dataset and Information Gain for the phosphorylation and ubiquitylation datasets. We used the SVM implemented in the R package “kernlab” [30] as the classifier for all three final predictive models, based on (i) performance measures (previous section), (ii) cross-platform compatibility, and (iii) ease of computational coding. These models were then implemented as an R package which is available at http://sysbio.chula.ac.th/PtmPPIR.

The final models were evaluated using 10 independent iterations of 5-fold cross-validation, and the resulting calculated performance measures for the models were high (Table 4). Nevertheless, to independently confirm the robustness of our models, an ideal approach would be to test the models using sequences with known localization in relation to PPIRs but which were not previously used in generation of the models. Therefore, we collected additional non-overlapped sequences from dbPTM [9], Huebner et al. [31], and Hou et al. [32] to construct the validation datasets for acetylation, phosphorylation, and ubiquitylation that reflected reality, i.e., possessing large class imbalances (see Table 7 and Additional file 5: Table S12). Table 8 shows the results following predictions by the final models for the validation datasets. Overall, performance measures were relatively good: AUC for the predictions was ≥ 0.82 for all three PTM datasets, PPV (precision) was similar (≥0.79), and FPR was ≤ 4 %.

**Table 7:** The validation datasets of sequences collected from dbPTM [5], Huebner et al. [31], and Hou et al. [32]

Dataset	Inside PPIRs	Outside PPIRs	Total
Acetylation	14	71	85
Phosphirylation	92	542	634
Ubiquitylation	33	71	104

**Table 8:** Results of model evaluation using the validation dataset

Dataset	PPV	F_1_	S_n_	S_p_	FPR	FNR	ACC	AUC	MCC
Acetylation	0.82	0.72	0.64	0.97	0.03	0.36	0.92	0.91	0.68
Phosphirylation	0.79	0.81	0.84	0.96	0.04	0.16	0.94	0.93	0.78
Ubiquitylation	0.87	0.71	0.61	0.96	0.04	0.39	0.85	0.82	0.63

Finally, the overall performance of our predictive models was compared with that of NPS-HomPPI [33], a more general method for predicting protein interaction sites (i.e., without regard to presence or absence of PTMs). After employing our models and NPS-HomPPI to perform predictions for the validation datasets, our method outperformed NPS-HomPPI (Additional file 6: Table S13), again indicating that the method described here is relatively good at identifying the characteristics of PTM sites on protein-protein interfaces.

### Prediction of PPIR localization for PTMs in the absence of 3D structure information

Finally, we applied our predictive models to the large number of sequences from the PhosphoSitePlus database that have no corresponding 3D structure in the PDB database (i.e., the ~ 380,000 PTM sequences with no mapped location inside/outside PPIRs). Table 9 shows the prediction results for these sequences with currently unknown PPIR localization. For the three PTMs studied here, 1–4 % of the modification sites were predicted to be located inside PPIRs.

**Table 9:** Results of predictions using sequences with unknown PPIR localization (see Additional file 10: Table S11 for the complete list of predicted PPIR localization for these sequences)

Dataset	Total # of sequences	Prediction result			
		Inside PPIRs	Percent	Outside PPIRs	Percent
Acetylation	32033	359	1.1	31674	98.9
Phosphorylation	258407	8967	3.5	249440	96.5
Ubiquitylation	49628	411	0.8	49217	99.2

## Discussion

In this study, we developed machine learning models for predicting whether post-translationally modified sites in proteins are inside or outside of PPIRs (protein-protein interacting regions). We combined data from two of the most comprehensive databases currently available, namely 3D structural and protein-protein interaction data from the PDB, and protein modification site data from the PhosphoSitePlus database. After the filtering and mapping processes (Fig. 2), only the three most abundant types of modifications (acetylation, phosphorylation, and ubiquitylation) had sufficient numbers of modification sites (≥500) for further analyses. In addition, the number of interacting residues in the final PPIR map represented only a minor fraction (average of ~15 %) of the total residues present in PDB polypeptide chains, and only 16 % of the known PTM sites (from the PhosphoSitePlus database) were located inside PPIRs. Despite these limitations which reduced the number of sequences available for analysis, the final predictive models generated in this study were characterized by relatively high performance measures. These results were in contrast to those obtained during our initial attempts to generate such predictive models, using either existing web-based applications or a machine learning approach based on a limited set of conventional features (see Table 1).

During the initial generation of the predictive models, the imbalance between the total numbers of interacting and non-interacting sites caused misclassifying during the learning process, as indicated by poor performance measures (Additional file 7: Table S1, Additional file 1: Table S2). In addition, computational times required for generating predictive models were relatively long for the imbalanced datasets (Table 10). After balancing by under-sampling techniques using a clustering algorithm, performance measures were significantly improved (Table 2 compared to Additional file 1: Table S2) and computational times were decreased approximately 10-fold (Table 10). Besides under-sampling techniques, over-sampling techniques also have been used to cope with imbalanced datasets [34]. However, it has been reported that certain over-sampling techniques may lead to over-fitting, a phenomenon in which the resulting models perform well with the training dataset but subsequently exhibit poor performance measures during the validation process [35].

**Table 10:** Relative computational time required for generating predictive models

Dataset	Computational time required (ms)	
	Balanced dataset	Imbalanced dataset
Acetylation	4,304	38,872
Phosphorylation	37,840	351,856
Ubiquitylation	12,009	124,460

All tests were performed on a personal computer (CPU:Intel®Core™i5-4200U, RAM:8.00 GB, OS:Windows7 Ultimate)

The initial predictive models were generated using 102 individual indices from AAindex1 and then refined by index ranking and feature selection processes to identify the optimal reduced set of indices and features for each PTM-specific dataset (see Methods). For the initial collection of indices from AAindex1, approximately half were related to hydrophobic properties of the amino acids; after optimization, a similar representation of hydrophobicity-related indices was observed for each PTM-specific dataset, as well as for the overlapping set of 20 indices common to all three PTMs (see Fig. 3). This finding is not surprising and is consistent with previous studies demonstrating the importance of hydrophobicity to protein-protein interactions [36, 37]. Nevertheless, no single property can distinguish between residues located inside and outside of PPIRs, and most methods for predicting residues at protein-protein interfaces use a combination of several properties [38]. Furthermore, factors other than hydrophobicity were also important to the performance of the models presented here—and presumably, to the actual protein-protein interaction themselves—as evident from the values of performance measures determined separately for the hydrophobicity- and non-hydrophobicity-related indices (see Additional file 8: Table S7).

While the final predictive models generated in this study were characterized by relatively high performance measures, one limitation needs to be mentioned. Independent evaluation of the models’ performance was only possible for PTMs located outside PPIRs, since all the PTMs known to be located inside PPIRs were used to generate the models. Thus, the high level of predicted accuracy observed for PTMs located outside PPIRs (≥90 %) should not be directly extrapolated to the other class of PTMs (located inside PPIRs). Nevertheless, when the models were applied to the large number of PTM sequences with no PDB structure information (~380,000), between 1–4 % of the modification sites were predicted to be located inside PPIRs (Table 9). As already mentioned above, ~16 % of the mapped PTM sites with known 3D structures were actually located inside PPIRs (3693 out of 22,482; see Fig. 1). The apparent discrepancy between this latter value for sites with known structure (16 %) and the value of 1–4 % for sites lacking structure information can be explained. Proteins with no interacting partners were removed from the downloaded dataset of PDB structures prior to mapping PTM sites. Therefore, the mapped PTM sites were enriched with interacting sequences relative to the broader population of PTM sequences with unknown structure.

We anticipate that the predictive models presented here will be useful in several ways. First, as large numbers of new PTMs are identified using high-throughput proteomics techniques (e.g., LC-MS/MS), it will be necessary to prioritize which individual PTMs will be selected for further studies of their roles in regulating protein functions. The prediction for any newly identified PTMs as being localized inside PPIRs would help with this prioritization, since PTMs located inside PPIRs are likely to significantly impact (promote or disrupt) protein-protein interactions. However, we should note one caveat to this generalized approach in predicting the importance of specific PTMs for their effects on protein-protein interactions: some PTMs located outside of PPIRs could also affect protein-protein interactions (e.g., by inducing a conformational change, etc.), but such PTMs would not be identified using our approach. As a second potential use for the data generated by these models, investigators can search the lists provided here of known PTMs either determined or predicted to be located inside PPIRs (Additional files 9 and 10, respectively). Such searches for specific PTM sites in proteins of interest could facilitate prioritization for further functional studies. As one final note, since our tool was designed and trained based on sites of known modifications, it should not be used for predictions of sites for which modification status is unknown.

## Conclusion

In this study, we developed the first models for predicting whether sites of protein modifications are inside or outside of protein-protein interacting regions (PPIRs), based on the existing structural and PTM databases. Our models show relatively high predictive performance measures. As more data become available, the performance of these models should be even better. Specifically, discovery of new PPIRs as a result of rapidly increasing 3D structure determinations should increase the accuracy of the predictive models. These predictive models are available online and may be useful in providing additional insight related to the functional relationship between specific PTMs and protein-protein interactions.

## Methods

### Generation and preparation of datasets

The protein-protein interacting region (PPIR) map was generated based on the PDB database [15]. First, all PDB structures were downloaded from the PDB database (10/19/2014) using FTP service (ftp://ftp.wwpdb.org/). Because a significant number of PDB files remain uncurated, it was necessary to use filtering software (written in-house) to remove files with unusable formats (e.g., files containing mislabeled chains, multiple conformational states, lack of sequence agreement with the corresponding Uniprot ID, etc.). Subsequently, an additional filtering software module (also written in-house) was employed to remove those PDB files for proteins with no interacting protein partners. Next, the dataset containing the remaining PDB files was analyzed using the “contact” function of Jmol [39] to detect specific residues that form contact points between interacting protein partners (i.e., between both heterologous and homologous polypeptide chains, but not between interfaces on a single polypeptide chain). For this analysis, a pair of interacting residues is defined as residues with an overlapped Van der Waals surface. A modified site was included in our PPIR map when it was determined from Jmol calculations to be a contact residue in at least one PPIR. For proteins with multiple interacting partners, since all subsequent analyses were based on the sequences containing the modified sites, the number and identity of PPI partner(s) became irrelevant. Thus, the final map contained only non-redundant information for each modified site, independent of its binding partners. The assignment of every residue in the dataset as interacting or non-interacting was recorded to produce the PPIR map.

Datasets of protein post-translational modifications (PTMs) were downloaded from PhosphoSitePlus (3/29/2015) [12]. These datasets consist of peptide sequences that are up to 15 amino acid residues in length and which include the modification site and up to seven neighboring amino acids on either side of the site. In a few cases (<1 %), the modification sites were close to protein termini and thus, the length of sequence was less than 15. To facilitate the subsequent computational analyses, these sequences were removed from the datasets. During construction of our models we examined the effect of sequence length (3–15 residues) on performance and found that a sequence length of 15 provided the highest performance measures (data provided to reviewer but not shown here). Employing a matching algorithm (written in-house), the remaining sequences were then used as input for the interacting residue map (generated from the PDB database as described above) to find an exact match in the PPIR map for each PTM sequence; simultaneously, sequences with no exact match, representing those with no corresponding structure information in the PDB database, were removed. The mapped PTM sequences were identified and tagged as being inside or outside of a PPIR, based on whether the modified residues were assigned as interacting or non-interacting in the PPIR map. After the modified sites were identified as being inside or outside of a PPIR, sequences were segregated into seven PTM-specific datasets, corresponding to the seven different types of modifications represented in the PhosphoSitePlus database (i.e., acetylation, methylation, O-GalNAc, O-GlcNAc, phosphorylation, sumoylation, and ubiquitylation) (see Additional file 9: Table S10 for the three datasets analyzed further in this study).

### Analysis of datasets using conventional features

PTM–specific datasets were subjected to analysis using different existing algorithms to calculate position-specific amino acid preferences for the modified site and its surrounding residues: PhosphoLogo [24] (https://hpcwebapps.cit.nih.gov/PhosphoLogo/) was used for the phosphorylation dataset, and Motif-x [25] (http://motif-x.med.harvard.edu/motif-x.html) was employed for the acetylation and ubiquitylation datasets. Please note that PhosphoLogo is not compatible with phosphorylated histidine input [24]. Therefore, sequences corresponded to phosphorylated histidine were removed before using PhosphoLogo. These sequences were belonged to bacteria species. Additional file 11: Figure S1 showed the numbers of sequence in acetylation, phosphorylation, and ubiquitylation datasets categorized by their source organisms. Three additional features were also subjected to the classification task of the machine leaning strategy; hydropathy indices were calculated using Kyte-Doolittle hydropathy scores executed in R [40]; secondary structure analysis was performed by NetsurfP [41] (http://www.cbs.dtu.dk/services/NetSurfP/); conservation of the modified site and its surrounding residues was calculated by CPhos [42] (https://hpcwebapps.cit.nih.gov/CPhos/).

### Sampling strategy for imbalanced datasets

Based on the PPIR map, the number of interacting residues (average of ~15 % of all residues) was markedly smaller than that of non-interacting residues. Therefore, the chance for a modified site to be located inside an interacting region was correspondingly lower than that for localization outside an interacting region, thus creating a class imbalance which introduces a computational bias. Such an imbalance causes misclassification during the learning process of predictive model generation [43–45]. In this specific study, machine learning algorithms would be overwhelmed by modified sites located outside an interacting region and would ignore those located inside an interacting region.

One solution to deal with this imbalance problem is an approach called under-sampling [46–48], using a clustering algorithm to equalize the number of interacting and non-interacting sequences. First, each PTM–specific dataset was categorized into interacting and non-interacting sub-datasets, then the non-interacting sub-dataset was clustered into 10 groups by GibbsCluster [26], based on position-specific scoring matrices (PSSM), in order to maintain proportional representation of relative sequence similarities in this sub-dataset. Finally, from each cluster we randomly selected an equivalent proportion of sequences, such that the combined size of the reduced non-interacting sub-dataset was approximately equal to that of the interacting sub-dataset. Figure 5 illustrates the strategy for balancing interacting and non-interacting sub-datasets in this study.

### Data encoding

After the balancing process, sequences contained in each of the seven PTM-specific datasets were then encoded into numerical data using AAindex1 [19], a database of 544 numerical indices representing various physicochemical and biochemical properties of amino acids. These indices can be grouped into 6 general categories representing different fundamental properties of amino acids (e.g., hydrophobicity, secondary structure probability, etc.) [19, 49].

A total of 102 indices (out of 544) were selected for use in the encoding step (see Additional file 12: Table S8) based on the following criteria: 1) since each general category of AAindex1 includes multiple indices for a specific property, only one index per specific property was chosen (i.e., indices were chosen to be non-redundant); 2) only indices representing amino acid properties directly related to protein structure were selected; and 3) indices representing each of the 6 general categories of fundamental amino acids properties were selected, with a final proportional representation of indices from each category similar to that of the entire database.

For each index, an individual sequence was first encoded into a vector of 15 numeric values. Next, values at each position 1–7 and 9–15 were subtracted from the value of position 8, which is the modification site, yielding a vector of 14 numeric values. Thus, in total, each sequence was encoded into a vector of 1428 values, since 102 indices were used. For each PTM-specific dataset, all vectors were concatenated into an array, wherein each row represented an individual sequence and each column represented a position-specific feature.

### Classification task

The support vector machine (SVM) has been widely applied in computational biology fields due to its high predictive performance [50–53] compared to other classifiers. Therefore, encoded PTM-specific datasets were used as input for the SVM using the R package “kernlab” [30]. Throughout this study, the parameter C (cost of constraint violation), epsilon, and the type of kernel used for the SVM were 1, 0.1, and the radial basis kernel, respectively. The predictive performance measures were evaluated using 10 independent iterations of 5-fold cross-validation. Here, the predictive performance measures evaluated were averaged values of overall accuracy (ACC), Matthew’s correlation coefficient (MCC), precision or positive predictive value (PPV), F-measure (F_1_), sensitivity (S_n_) or true positive rate (TPR), specificity (S_p_) or true negative rate (TNR), and area under receiver operating characteristic curve (AUC). ACC, MCC, PPV, F_1_, S_n_, and S_p_ are defined as

$$ ACC=\frac{TP+TN}{TP+TN+FP+FN} $$

$$ MCC=\frac{\left(TP\times TN\right)-\left(FP\times FN\right)}{\sqrt{\left(TP+FN\right)\left(TP+FP\right)\left(TN+FP\right)\left(TN+FN\right)}} $$

$$ PPV=\frac{TP}{\left(TP+FP\right)} $$

$$ {F}_1=\frac{2TP}{\left(2TP+FP+FN\right)} $$

$$ {S}_n=\frac{TP}{\left(TP+FN\right)} $$

$$ {S}_p=\frac{TN}{\left(FP+TN\right)} $$

where TP, FP, TN, and FN are the number of overall true positives, false positives, true negatives, and false negatives, respectively. For this study, the definitions of TP, FP, TN, and FN are shown in Additional file 13: Table S9.

### Feature selection task

To improve the performance of the predictive models, optimal subsets of indices (from AAindex1) were identified for encoded PTM-specific datasets using a “greedy-based” algorithm. First, individual encoded dataset arrays were divided into 102 14-column blocks of features (a total of 1428 features), each block representing an individual index. The classification functions were fitted to these index-specific sub-datasets, and indices were then ranked based on multiple performance measures. While AUC is the most frequently used performance measure, alternative performance measures could also be used, each of which could potentially yield different results. Thus, to minimize biases arising from any individual measure, we employed an approach based on the highest summation of three different performance measures (AUC, ACC, and MCC), designated as the Combined Performance Score (CPS). After initial ranking of indices using CPS, classification tasks were performed sequentially by including the index-specific sub-datasets one by one in order of their individual rank. As a result, the optimal set of indices that led to the maximum CPS value for each PTM-specific dataset was identified. Finally, encoded PTM-specific datasets were reduced to contain only sub-datasets corresponding to indices in the optimal sets.

For each reduced PTM-specific dataset, the Relief-F [27] and Information Gain [28] algorithms, implemented in the machine learning software Weka [54], were employed to rank the features. The default parameters provided by Weka were used for evaluating feature importance. The best-feature subsets were constructed by adding the features sequentially, one by one, from the top ranked feature to the last one in the classification task using the SVM. The CPS gradually increased with the addition of features, until it reached the maximum value. Features after this point were considered irrelevant and ignored. The resulting reduced feature subsets were then used in all subsequent analyses.

### Comparisons of classifiers

The reduced feature subsets for encoded PTM-specific datasets were evaluated in the classification tasks using the k-nearest neighbors (k-NN, k = 10), KStar, Random Forest (RF), C4.5, and Multilayer Perceptron (MLP) classifiers implemented in the machine learning software Weka [54]. The default parameters provided by Weka were used in classification tasks which were conducted in 10 independent iterations of 5-fold cross-validation. The results of performance measures were then compared among classifiers.

### Final predictive models

The classifier that led to the highest performance measures was used to generate final predictive models that were specific to each PTM type. The R language was used to implement the models. We host the R package in our server at http://sysbio.chula.ac.th/PtmPPIR.

## Abbreviations

AUC, area under receiver operating characteristic curve; CPS, combined performance score; k-NN, k-nearest neighbors; MCC, Matthew’s correlation coefficient; MLP, multilayer perceptron; PPIR, protein-protein interacting region; PTM, post-translational modification; RF, random forest

## Additional files

Additional file 1: Table S2. Averaged performance measures of three PTM-specific datasets when using the SVM as a classifier and 102 indices of AAindex1 in the encoding process. (DOCX 18 kb) Additional file 2: Table S3. Optimized set of indices for acetylation dataset. (DOCX 25 kb) Additional file 3: Table S4. Optimized set of indices for phosphorylation dataset. (DOCX 21 kb) Additional file 4: Table S5. Optimized set of indices for ubiquitylation dataset. (DOCX 26 kb) Additional file 5: Table S12. Lists of sequences in the validation datasets. (XLSX 46 kb) Additional file 6: Table S13 Comparison of predictive performance between our method and NPS-HomPPI for the validation datasets. (DOCX 30 kb) Additional file 7: Table S1. Classification results of imbalanced-three PTM-specific datasets when conventional features were used and the SVM is employed as a classifier. (DOCX 21 kb) Additional file 8: Table S7. Performance comparisons for the SVM using different selected sets of indices. Numbers in the parentheses indicate number of indices used in each analysis. (DOCX 22 kb) Additional file 9: Table S10. List of known PTMs identified to be located inside/outside PPIRs after mapping process. (XLSX 677 kb) Additional file 10: Table S11. List of known PTMs predicted to be located inside/outside PPIRs by using our predictive models. (XLSX 9201 kb) Additional file 11: Figure S1. The number of sequences categorized by their source organisms. (PDF 837 kb) Additional file 12: Table S8. 102 indices of AAindex1used in this study. (DOCX 25 kb) Additional file 13: Table S9. Definitions of true positives (TP), false positives (FP), true negatives (TN), and false negatives (FN) in this study. (DOCX 18 kb) Additional file 14: Table S6. Common overlapping indices of three PTM-specific optimized sets of indices. (DOCX 20 kb)
