# Supplementary material for: A machine learning strategy for predicting localization of post-translational modification sites in protein-protein interacting regions
Source: BMC Bioinformatics. 2016 Aug 17;17:307. doi: 10.1186/s12859-016-1165-8 (PMC4989344; doi:10.1186/s12859-016-1165-8)
Supplement: Additional file 1: Table S2. — Averaged performance measures of three PTM-specific datasets when using the SVM as a classifier and 102 indices of AAindex1 in the encoding process. (DOCX 18 kb) [file 12859_2016_1165_MOESM1_ESM.docx]

**Table S2** Averaged performance measures of three PTM-specific datasets when using the SVM as a classifier and 102 indices of AAindex1 in the encoding process.

| **Dataset** | **F_1_** | **TPR** | **SPC** | **ACC** | **AUC** | **MCC** |
| --- | --- | --- | --- | --- | --- | --- |
| Acetylation | NaN | 0.00 | 1.00 | 0.83 | 0.48 | NaN |
| Phosphorylation | NaN | 0.00 | 1.00 | 0.84 | 0.54 | NaN |
| Ubiquitylation | NaN | 0.00 | 1.00 | 0.82 | 0.52 | NaN |

The highest value for all measures is 1.
Standard deviations are less than 0.005.
