# Supplementary material for: A machine learning strategy for predicting localization of post-translational modification sites in protein-protein interacting regions
Source: BMC Bioinformatics. 2016 Aug 17;17:307. doi: 10.1186/s12859-016-1165-8 (PMC4989344; doi:10.1186/s12859-016-1165-8)
Supplement: Additional file 2: Table S3. — Optimized set of indices for acetylation dataset. (DOCX 25 kb) [file 12859_2016_1165_MOESM2_ESM.docx]

**Table S3** Optimized set of indices for acetylation dataset

| Rank | AAindex1 | Accession number [15] |
| --- | --- | --- |
| 1 | Normalized frequency of N-terminal beta-sheet | CHOP780208 |
| 2 | Optimal matching hydrophobicity | SWER830101 |
| 3 | A parameter of charge transfer capability | CHAM830107 |
| 4 | Normalized frequency of beta-sheet | CHOP780202 |
| 5 | Hydrophobicity factor | GOLD730101 |
| 6 | Normalized hydrophobicity scales for beta-proteins | CIDH920102 |
| 7 | Normalized average hydrophobicity scales | CIDH920105 |
| 8 | Negative charge | FAUJ880112 |
| 9 | Polarity | GRAR740102 |
| 10 | Net charge | KLEP840101 |
| 11 | Isoelectric point | ZIMJ680104 |
| 12 | Normalized hydrophobicity scales for alpha/beta-proteins | CIDH920104 |
| 13 | Normalized hydrophobicity scales for alpha-proteins | CIDH920101 |
| 14 | Signal sequence helical potential | ARGP820102 |
| 15 | Solvation free energy | EISD86010 |
| 16 | Hydrophilicity value | HOPT810101 |
| 17 | Hydration number | HOPA770101 |
| 18 | Average flexibility indices | BHAR880101 |
| 19 | Normalized frequency of beta-sheet | CRAJ730102 |
| 20 | Normalized frequency of N-terminal helix | CHOP780204 |
| 21 | Transfer free energy, CHP/water | LAWE840101 |
| 22 | Membrane-buried preference parameters | ARGP820103 |
| 23 | Normalized hydrophobicity scales for alpha+beta-proteins | CIDH920103 |
| 24 | Hydrophobic parameter | LEVM760101 |
| 25 | Hydrophobic parameter pi | FAUJ830101 |
| 26 | Hydrophilicity scale | KUHL950101 |
| 27 | Normalized frequency of extended structure | BURA740101 |
| 28 | Buriability | ZHOH040103 |
| 29 | pK-N | FASG760104 |
| 30 | Hydrophobicity index | ENGD860101 |
| 31 | Hydrophobicity | PRAM900101 |
| 32 | pK-a(RCOOH) | FAUJ880113 |
| 33 | Average gain ratio in surrounding hydrophobicity | PONP800103 |
| 34 | Mean polarity | RADA880108 |
| 35 | Hydrophobicity scales | PONP930101 |
| 36 | Polarity | ZIMJ680103 |
| 37 | Bulkiness | ZIMJ680102 |
| 38 | Hydropathy index | KYTJ820101 |
| 39 | Normalized frequency of C-terminal beta-sheet | CHOP780209 |
| 40 | Information value for accessibility; average fraction 23% | BIOV880102 |
| 41 | Information value for accessibility; average fraction 35% | BIOV880101 |
| 42 | Relative partition energies derived by the Bethe approximation | MIYS990101 |
| 43 | Direction of hydrophobic moment | EISD860103 |
| 44 | Localized electrical effect | FAUJ880108 |
| 45 | Free energy of solution in water, kcal/mole | CHAM820102 |
| 46 | Modified Kyte-Doolittle hydrophobicity scale | JURD980101 |
| 47 | 8 A contact number | NISK800101 |
| 48 | Hydrophobicity index | ARGP820101 |
| 49 | Hydrophobicity | JOND750101 |
| 50 | Optical rotation | FASG760103 |
| 51 | Average gain in surrounding hydrophobicity | PONP800102 |
| 52 | Hydrophobicity-related index | KIDA850101 |
| 53 | Transfer free energy | JANJ790102 |
| 54 | Consensus normalized hydrophobicity scale | EISD840101 |
| 55 | Mean fractional area loss | ROSG850102 |
| 56 | Helix formation parameters | ONEK900102 |
| 57 | Normalized frequency of beta-turn | CHOP780203 |
| 58 | Surrounding hydrophobicity in folded form | PONP800101 |
| 59 | Partition energy | GUYH850101 |
| 60 | Partition coefficient | PLIV810101 |
| 61 | Hydration potential | WOLR810101 |
| 62 | Hydration free energy | ROBB790101 |
| 63 | Normalized frequency of C-terminal non beta region | CHOP780211 |
| 64 | Hydrophobicity index | FASG890101 |
| 65 | Hydrophobicity index | WOLR790101 |
| 66 | Percentage of buried residues | JANJ780102 |
| 67 | Normalized frequency of N-terminal non beta region | CHOP780210 |
| 68 | Ratio of buried and accessible molar fractions | JANJ790101 |
| 69 | Mean area buried on transfer | ROSG850101 |
| 70 | Melting point | FASG760102 |
| 71 | A parameter defined from the residuals obtained from the best correlation of the Chou-Fasman parameter of beta-sheet | CHAM830102 |
