# Supplementary material for: A machine learning strategy for predicting localization of post-translational modification sites in protein-protein interacting regions
Source: BMC Bioinformatics. 2016 Aug 17;17:307. doi: 10.1186/s12859-016-1165-8 (PMC4989344; doi:10.1186/s12859-016-1165-8)
Supplement: Additional file 3: Table S4. — Optimized set of indices for phosphorylation dataset. (DOCX 21 kb) [file 12859_2016_1165_MOESM3_ESM.docx]

**Table S4** Optimized set of indices for phosphorylation dataset

| Rank | AAindex1 | Accession number [15] |
| --- | --- | --- |
| 1 | Signal sequence helical potential | ARGP820102 |
| 2 | Normalized hydrophobicity scales for beta-proteins | CIDH920102 |
| 3 | A parameter of charge transfer donor capability | CHAM830108 |
| 4 | Free energy of solution in water, kcal/mole | CHAM820102 |
| 5 | Steric parameter | CHAM810101 |
| 6 | Hydrophobicity factor | GOLD730101 |
| 7 | Melting point | FASG760102 |
| 8 | Optimal matching hydrophobicity | SWER830101 |
| 9 | Normalized average hydrophobicity scales | CIDH920105 |
| 10 | Polarity | GRAR740102 |
| 11 | Amphiphilicity index | MITS020101 |
| 12 | Buriability | ZHOH040103 |
| 13 | Polarizability parameter | CHAM820101 |
| 14 | Relative partition energies derived by the Bethe approximation | MIYS990101 |
| 15 | Accessible surface area | RADA880106 |
| 16 | Normalized frequency of beta-sheet | CHOP780202 |
| 17 | Residue accessible surface area in folded protein | CHOC760102 |
| 18 | Normalized hydrophobicity scales for alpha-proteins | CIDH920101 |
| 19 | Hydrophobicity index | ARGP820101 |
| 20 | Normalized frequency of beta-sheet | CRAJ730102 |
| 21 | Hydrophobicity | JOND750101 |
| 22 | Absolute entropy | HUTJ700102 |
| 23 | A parameter defined from the residuals obtained from the best correlation of the Chou-Fasman parameter of beta-sheet | CHAM830102 |
| 24 | Normalized van der Waals volume | FAUJ880103 |
| 25 | Bulkiness | ZIMJ680102 |
| 26 | Normalized frequency of middle helix | CRAJ730101 |
| 27 | A parameter of charge transfer capability | CHAM830107 |
| 28 | Hydration free energy | ROBB790101 |
| 29 | Average volume of buried residue | CHOC750101 |
| 30 | Normalized hydrophobicity scales for alpha/beta-proteins | CIDH920104 |
| 31 | Normalized frequency of N-terminal non helical region | CHOP780206 |
