# Supplementary material for: A machine learning strategy for predicting localization of post-translational modification sites in protein-protein interacting regions
Source: BMC Bioinformatics. 2016 Aug 17;17:307. doi: 10.1186/s12859-016-1165-8 (PMC4989344; doi:10.1186/s12859-016-1165-8)
Supplement: Additional file 6: Table S13 — Comparison of predictive performance between our method and NPS-HomPPI for the validation datasets. (DOCX 30 kb) [file 12859_2016_1165_MOESM6_ESM.docx]

**Table S13** Comparison of predictive performance between our method and NPS-HomPPI* for the validation datasets

|  | NPS-HomPPI | | | | | | | |
| --- | --- | --- | --- | --- | --- | --- | --- | --- |
| Dataset | PPV | F_1_ | S_n_(TPR) | S_p_(TNR) | FPR | FNR | ACC | MCC |
| Acetylation | 0.44 | 0.35 | 0.29 | 0.93 | 0.07 | 0.71 | 0.82 | 0.26 |
| Phosphorylation | 0.45 | 0.45 | 0.46 | 0.90 | 0.10 | 0.54 | 0.84 | 0.36 |
| Ubiquitylation | 0.65 | 0.44 | 0.33 | 0.92 | 0.08 | 0.67 | 0.73 | 0.31 |
|  | **Our method** | | | | | | | |
| Acetylation | 0.82 | 0.72 | 0.64 | 0.97 | 0.03 | 0.36 | 0.92 | 0.68 |
| Phosphorylation | 0.79 | 0.81 | 0.84 | 0.96 | 0.04 | 0.16 | 0.94 | 0.78 |
| Ubiquitylation | 0.87 | 0.71 | 0.61 | 0.96 | 0.04 | 0.39 | 0.85 | 0.63 |

**NOTE** Sensitivity = true positive rate (TPR); Specificity = true negative rate (TNR);
Precision = positive predictive value (PPV)

* L.C. Xue, D. Dobbs, V. Honavar. HomPPI: a class of sequence homology based protein–protein interface prediction methods. BMC Bioinformatics, 12 (1) (2011), p. 244 <http://dx.doi.org/10.1186/1471-2105-12-244>
