# Supplementary material for: A machine learning strategy for predicting localization of post-translational modification sites in protein-protein interacting regions
Source: BMC Bioinformatics. 2016 Aug 17;17:307. doi: 10.1186/s12859-016-1165-8 (PMC4989344; doi:10.1186/s12859-016-1165-8)
Supplement: Additional file 7: Table S1. — Classification results of imbalanced-three PTM-specific datasets when conventional features were used and the SVM is employed as a classifier. (DOCX 21 kb) [file 12859_2016_1165_MOESM7_ESM.docx]

**Table S1** Classification results of imbalanced**-**three PTM-specific datasets when conventional features were used and the SVM is employed as a classifier.

|  | F_1_ | TPR | SPC | ACC | AUC | MCC |
| --- | --- | --- | --- | --- | --- | --- |
| Acetylation |  |  |  |  |  |  |
| Hydropathy | NaN | 0.00 | 1.00 | 0.83 | 0.50 | NaN |
| 2^nd^ structure | NaN | 0.00 | 1.00 | 0.83 | 0.47 | NaN |
| Conservation | NaN | 0.00 | 1.00 | 0.81 | 0.52 | NaN |
| Combined features | NaN | 0.00 | 1.00 | 0.81 | 0.52 | NaN |
| Phosphorylation |  |  |  |  |  |  |
| Hydropathy | NaN | 0.00 | 1.00 | 0.84 | 0.50 | NaN |
| 2^nd^ structure | 0.10 | 0.05 | 1.00 | 0.84 | 0.50 | 0.01 |
| Conservation | NaN | 0.00 | 1.00 | 0.85 | 0.51 | NaN |
| Combined features | NaN | 0.00 | 1.00 | 0.85 | 0.51 | NaN |
| Ubiquitylation |  |  |  |  |  |  |
| Hydropathy | NaN | 0.00 | 1.00 | 0.82 | 0.50 | -0.01 |
| 2^nd^ structure | NaN | 0.00 | 1.00 | 0.82 | 0.50 | NaN |
| Conservation | NaN | 0.00 | 1.00 | 0.83 | 0.53 | NaN |
| Combined features | NaN | 0.00 | 1.00 | 0.83 | 0.51 | NaN |
