# Supplementary material for: A machine learning strategy for predicting localization of post-translational modification sites in protein-protein interacting regions
Source: BMC Bioinformatics. 2016 Aug 17;17:307. doi: 10.1186/s12859-016-1165-8 (PMC4989344; doi:10.1186/s12859-016-1165-8)
Supplement: Additional file 8: Table S7. — Performance comparisons for the SVM using different selected sets of indices. Numbers in the parentheses indicate number of indices used in each analysis. (DOCX 22 kb) [file 12859_2016_1165_MOESM8_ESM.docx]

**Table S7** Performance comparisons for the SVM using different selected sets of indices. Numbers in the parentheses indicate number of indices used in each analysis.

|  | Acetylation | | | | | Phosphorylation | | | | | Ubiquitylation | | | | |
| --- | --- | --- | --- | --- | --- | --- | --- | --- | --- | --- | --- | --- | --- | --- | --- |
|  | **All (102)** | **Optimized (71)** | **Intersect (20)** | **Hydrophobicity**  **(11)** | **Non-hydrophobicity**  **(9)** | **All (102)** | **Optimized (71)** | **Intersect (20)** | **Hydrophobicity**  **(11)** | **Non-hydrophobicity**  **(9)** | **All (102)** | **Optimized (71)** | **Intersect (20)** | **Hydrophobicity**  **(11)** | **Non-hydrophobicity**  **(9)** |
| F1 | 0.86 | 0.87 | 0.87 | 0.80 | 0.85 | 0.84 | 0.89 | 0.89 | 0.79 | 0.87 | 0.86 | 0.86 | 0.87 | 0.83 | 0.85 |
| SPC | 0.94 | 0.93 | 0.94 | 0.90 | 0.94 | 0.99 | 1.00 | 1.00 | 1.00 | 1.00 | 0.92 | 0.92 | 0.96 | 0.83 | 0.96 |
| TPR | 0.81 | 0.82 | 0.81 | 0.73 | 0.78 | 0.74 | 0.81 | 0.81 | 0.65 | 0.78 | 0.81 | 0.81 | 0.80 | 0.83 | 0.76 |
| ACC | 0.87 | 0.87 | 0.87 | 0.81 | 0.86 | 0.86 | 0.90 | 0.90 | 0.83 | 0.89 | 0.86 | 0.87 | 0.87 | 0.83 | 0.86 |
| AUC | 0.89 | 0.90 | 0.89 | 0.88 | 0.90 | 0.92 | 0.92 | 0.92 | 0.83 | 0.92 | 0.90 | 0.90 | 0.90 | 0.89 | 0.89 |
| MCC | 0.75 | 0.76 | 0.75 | 0.63 | 0.73 | 0.75 | 0.82 | 0.82 | 0.69 | 0.79 | 0.73 | 0.74 | 0.76 | 0.66 | 0.73 |
| CPS | 2.51 | 2.53 | 2.51 | 2.32 | 2.48 | 2.53 | 2.64 | 2.64 | 2.35 | 2.60 | 2.49 | 2.51 | 2.53 | 2.37 | 2.48 |
