# Supplementary material for: A machine learning strategy for predicting localization of post-translational modification sites in protein-protein interacting regions
Source: BMC Bioinformatics. 2016 Aug 17;17:307. doi: 10.1186/s12859-016-1165-8 (PMC4989344; doi:10.1186/s12859-016-1165-8)
Supplement: Additional file 11: Figure S1. — The number of sequences categorized by their source organisms. (PDF 837 kb) [file 12859_2016_1165_MOESM11_ESM.pdf]

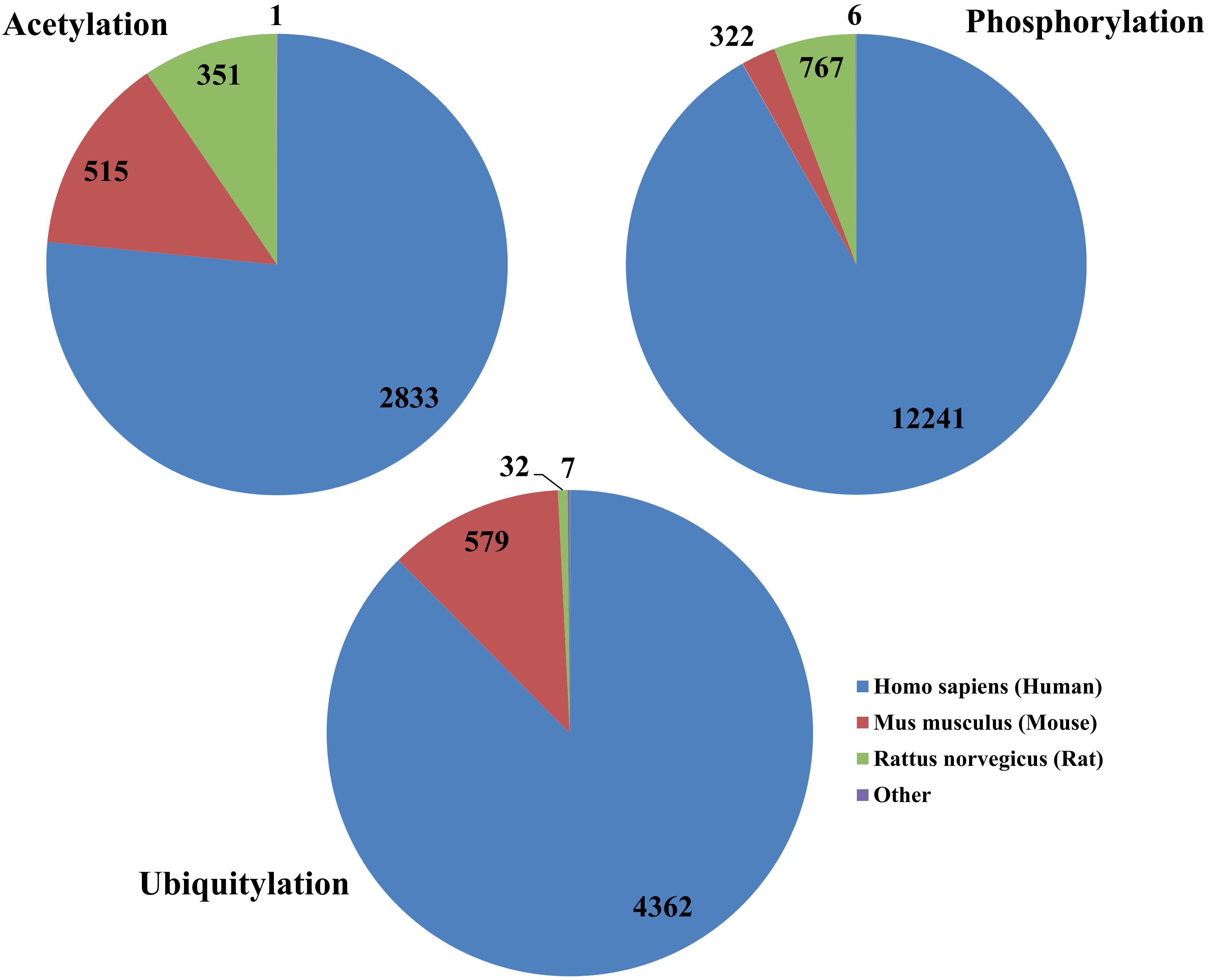
Acetylation
1
351
515
2833
Phosphorylation
322
6
767
12241
32
7
579
Ubiquitylation
4362
Homo sapiens (Human)
Mus musculus (Mouse)
Rattus norvegicus (Rat)
Other
