# Supplementary material for: A machine learning strategy for predicting localization of post-translational modification sites in protein-protein interacting regions
Source: BMC Bioinformatics. 2016 Aug 17;17:307. doi: 10.1186/s12859-016-1165-8 (PMC4989344; doi:10.1186/s12859-016-1165-8)
Supplement: Additional file 12: Table S8. — 102 indices of AAindex1used in this study. (DOCX 25 kb) [file 12859_2016_1165_MOESM12_ESM.docx]

**Table S8** 102 indices of AAindex1used in this study

| Accession number [15] | Indices |
| --- | --- |
| ARGP820101 | Hydrophobicity index |
| ARGP820102 | Signal sequence helical potential |
| ARGP820103 | Membrane-buried preference parameters |
| BHAR880101 | Average flexibility indices |
| BIGC670101 | Residue volume |
| BIOV880101 | Information value for accessibility; average fraction 35% |
| BIOV880102 | Information value for accessibility; average fraction 23% |
| BURA740101 | Normalized frequency of extended structure |
| CHAM810101 | Steric parameter |
| CHAM820101 | Polarizability parameter |
| CHAM820102 | Free energy of solution in water, kcal/mole |
| CHAM830101 | The Chou-Fasman parameter of the coil conformation |
| CHAM830102 | A parameter defined from the residuals obtained from the best correlation of the Chou-Fasman parameter of beta-sheet |
| CHAM830107 | A parameter of charge transfer capability |
| CHAM830108 | A parameter of charge transfer donor capability |
| CHOC750101 | Average volume of buried residue |
| CHOC760101 | Residue accessible surface area in tripeptide |
| CHOC760102 | Residue accessible surface area in folded protein |
| CHOP780101 | Normalized frequency of beta-turn |
| CHOP780201 | Normalized frequency of alpha-helix |
| CHOP780202 | Normalized frequency of beta-sheet |
| CHOP780203 | Normalized frequency of beta-turn |
| CHOP780204 | Normalized frequency of N-terminal helix |
| CHOP780205 | Normalized frequency of C-terminal helix |
| CHOP780206 | Normalized frequency of N-terminal non helical region |
| CHOP780207 | Normalized frequency of C-terminal non helical region |
| CHOP780208 | Normalized frequency of N-terminal beta-sheet |
| CHOP780209 | Normalized frequency of C-terminal beta-sheet |
| CHOP780210 | Normalized frequency of N-terminal non beta region |
| CHOP780211 | Normalized frequency of C-terminal non beta region |
| CIDH920101 | Normalized hydrophobicity scales for alpha-proteins |
| CIDH920102 | Normalized hydrophobicity scales for beta-proteins |
| CIDH920103 | Normalized hydrophobicity scales for alpha+beta-proteins |
| CIDH920104 | Normalized hydrophobicity scales for alpha/beta-proteins |
| CIDH920105 | Normalized average hydrophobicity scales |
| CRAJ730101 | Normalized frequency of middle helix |
| CRAJ730102 | Normalized frequency of beta-sheet |
| CRAJ730103 | Normalized frequency of turn |
| EISD840101 | Consensus normalized hydrophobicity scale |
| EISD86010 | Solvation free energy |
| EISD860102 | Atom-based hydrophobic moment |
| EISD860103 | Direction of hydrophobic moment |
| FASG760101 | Molecular weight |
| FASG760102 | Melting point |
| FASG760103 | Optical rotation |
| FASG760104 | pK-N |
| FASG760105 | pK-C |
| FAUJ830101 | Hydrophobic parameter pi |
| FAUJ880103 | Normalized van der Waals volume |
| FAUJ880108 | Localized electrical effect |
| FAUJ880111 | Positive charge |
| FAUJ880112 | Negative charge |
| FAUJ880113 | pK-a(RCOOH) |
| GOLD730101 | Hydrophobicity factor |
| GOLD730102 | Residue volume |
| GRAR740102 | Polarity |
| GRAR740103 | Volume |
| GUYH850101 | Partition energy |
| HOPA770101 | Hydration number |
| HOPT810101 | Hydrophilicity value |
| HUTJ700101 | Heat capacity |
| HUTJ700102 | Absolute entropy |
| HUTJ700103 | Entropy of formation |
| JANJ780101 | Average accessible surface area |
| JANJ780102 | Percentage of buried residues |
| JANJ780103 | Percentage of exposed residues |
| JANJ790101 | Ratio of buried and accessible molar fractions |
| JANJ790102 | Transfer free energy |
| JOND750101 | Hydrophobicity |
| JOND750102 | pK(-COOH) |
| KLEP840101 | Net charge |
| KYTJ820101 | Hydropathy index |
| LAWE840101 | Transfer free energy, CHP/water |
| LEVM760101 | Hydrophobic parameter |
| NISK800101 | 8 A contact number |
| OOBM850105 | Optimized side chain interaction parameter |
| PLIV810101 | Partition coefficient |
| PONP800101 | Surrounding hydrophobicity in folded form |
| PONP800102 | Average gain in surrounding hydrophobicity |
| PONP800103 | Average gain ratio in surrounding hydrophobicity |
| PRAM900101 | Hydrophobicity |
| RADA880106 | Accessible surface area |
| RADA880108 | Mean polarity |
| ROBB790101 | Hydration free energy |
| ROSG850101 | Mean area buried on transfer |
| ROSG850102 | Mean fractional area loss |
| SWER830101 | Optimal matching hydrophobicity |
| WOLR810101 | Hydration potential |
| ZIMJ680102 | Bulkiness |
| ZIMJ680103 | Polarity |
| ZIMJ680104 | Isoelectric point |
| ONEK900102 | Helix formation parameters |
| MITS020101 | Amphiphilicity index |
| PONP930101 | Hydrophobicity scales |
| KUHL950101 | Hydrophilicity scale |
| JURD980101 | Modified Kyte-Doolittle hydrophobicity scale |
| ZHOH040103 | Buriability |
| WOLR790101 | Hydrophobicity index |
| KIDA850101 | Hydrophobicity-related index |
| MIYS990101 | Relative partition energies derived by the Bethe approximation |
| ENGD860101 | Hydrophobicity index |
| FASG890101 | Hydrophobicity index |
