# Supplementary material for: A machine learning strategy for predicting localization of post-translational modification sites in protein-protein interacting regions
Source: BMC Bioinformatics. 2016 Aug 17;17:307. doi: 10.1186/s12859-016-1165-8 (PMC4989344; doi:10.1186/s12859-016-1165-8)
Supplement: Additional file 13: Table S9. — Definitions of true positives (TP), false positives (FP), true negatives (TN), and false negatives (FN) in this study. (DOCX 18 kb) [file 12859_2016_1165_MOESM13_ESM.docx]

**Table S9** Definitions of true positives (TP), false positives (FP), true negatives (TN), and false negatives (FN) in this study

|  |  | Actual class of PTM site | |
| --- | --- | --- | --- |
|  |  | inside a PPIR | outside a PPIR |
| Predicted class of PTM site | inside a PPIR | TP | FP |
|  | outside a PPIR | FN | TN |
