# Supplementary material for: A machine learning strategy for predicting localization of post-translational modification sites in protein-protein interacting regions
Source: BMC Bioinformatics. 2016 Aug 17;17:307. doi: 10.1186/s12859-016-1165-8 (PMC4989344; doi:10.1186/s12859-016-1165-8)
Supplement: Additional file 14: Table S6. — Common overlapping indices of three PTM-specific optimized sets of indices. (DOCX 20 kb) [file 12859_2016_1165_MOESM14_ESM.docx]

**Table S6** Common overlapping indices of three PTM-specific optimized sets of indices.

| Overlapped indices | Accession number [15] |
| --- | --- |
| Optimal matching hydrophobicity | SWER830101 |
| A parameter of charge transfer capability | CHAM830107 |
| Normalized frequency of beta-sheet | CHOP780202 |
| Hydrophobicity factor | GOLD730101 |
| Normalized hydrophobicity scales for beta-proteins | CIDH920102 |
| Normalized average hydrophobicity scales | CIDH920105 |
| Polarity | GRAR740102 |
| Normalized hydrophobicity scales for alpha/beta-proteins | CIDH920104 |
| Normalized hydrophobicity scales for alpha-proteins | CIDH920101 |
| Signal sequence helical potential | ARGP820102 |
| Normalized frequency of beta-sheet | CHOP780202 |
| Buriability | ZHOH040103 |
| Bulkiness | ZIMJ680102 |
| Relative partition energies derived by the Bethe approximation | MIYS990101 |
| Free energy of solution in water, kcal/mole | CHAM820102 |
| Hydrophobicity index | ARGP820101 |
| Hydrophobicity | JOND750101 |
| Hydration free energy | ROBB790101 |
| Melting point | FASG760102 |
| A parameter defined from the residuals obtained from the best correlation of the Chou-Fasman parameter of beta-sheet | CHAM830102 |
